# Ultrastructure of the proboscis sensilla of ten species of butterflies (Insecta: Lepidoptera)

**DOI:** 10.1371/journal.pone.0214658

**Published:** 2019-03-28

**Authors:** Luyao Ma, Kai Hu, Pengde Li, Jiaqi Liu, Xiangqun Yuan

**Affiliations:** Key Laboratory of Plant Protection Resources and Pest Management, Ministry of Education, College of Plant Protection, Northwest A&F University, Yangling, Shaanxi, China; Chinese Academy of Agricultural Sciences Institute of Plant Protection, CHINA

## Abstract

The ultrastructure of the sensilla on the proboscis of ten species of butterflies, *Iphiclides podalirius*, *Parara guttata*, *Colias fieldii*, *Celastrina oreas*, *Sasakia charonda*, *Tirumala limniace*, *Acraea issoria*, *Stichophthalma neumogeni*, *Callerebia suroia*, and *Libythea celtis*, among five families were investigated using scanning electron microscopy. They were compared to reveal the morphological differences in the proboscis sensilla among these butterflies. Four distinct types of sensilla were found on the proboscis among these species. The types of proboscis sensilla of *I*. *podalirius* and *T*. *limniace* were sensilla chaetica, sensilla coeloconica, and sensilla basiconica. The types in the other eight species were sensilla chaetica, sensilla styloconica, and sensilla basiconica. The number of sensilla styloconica on the proboscis of non-flower-visiting species was greater than that of flower-visiting species.

## Introduction

Members of the Lepidoptera (butterflies and moths) use the proboscis to acquire nutrition, water and pheromonal precursors from diverse sources, including soils, floral and extrafloral nectars, fruits, saps, plant surfaces, sugary exudates of plant-feeding insects, animal bloods, carrions, dungs, sweats, tears and urines [1–3]. The proboscises of Lepidoptera have various types of sensilla that play important roles in host location and feeding behavior [4–7]. Insect sensilla have been extensively recorded on the antennae [7–11] and proboscises [12–16], and have also been found on maxillary palps, labial palps [10], tarsi [17, 18], wings and nota [19]. The proboscis sensilla in Lepidoptera can be divided into six types according to their external morphology: sensilla chaetica, sensilla basiconica, sensilla styloconica, sensilla coeloconica, sensilla filiformia and sensilla campaniformia [1, 20].

Unique structures of the sensilla often accompany unique functions in feeding behavior. The sensilla chaetica, which are widely spaced at the base of the galea, are bristle-shaped, small and pointed, and are considered to be mechanoreceptors that provide information about the width and depth of the tubular flower during flower-probing [1, 21, 22]. The sensilla basiconica consist of a dome or peg-shaped sensory cone and a flexible and shallow socket [1, 23], which act as contact chemosensilla involved in the behavioral control of nectar-sucking [21, 24]. Sensilla styloconica are composed of a variously shaped styli and shorter terminal sensory cones as combined chemo- and mechanosensory organs [1, 21, 25]. They are sensitive to certain mono- and oligosaccharides and a variety of other substances [26–28]. The sensilla coeloconica have a smooth sphere in the deep cavity of the galeal cuticle and hardly reach the surface of the galea, which is linked to the particular food-seizing techniques of certain species [16, 20]. Sensilla filiformia are hair-like, longer than sensilla chaetica, and have been demonstrated to be mechanosensitive [20, 29]. Sensilla campaniformia are dome-shaped organs at the galeal base [20].

Adults of most butterfly species visit flowers for food (nectar), and certain species mainly Nymphalidae feed on rotting food (such as exuded tree saps and rotting fruits) [5, 30, 31]. In this study, *Iphiclides podalirius*, *Parara guttata*, *Colias fieldii*, *Celastrina oreas*, *Tirumala limniace*, *Acraea issoria*, *Callerebia suroia* and *Libythea celtis* feed on nectars, while *Sasakia charonda* feeds on the sap of trees, and *Stichophthalma neumogeni* feeds on the fruits. In this paper, we compare the morphology of the proboscis and their sensilla in ten species of butterflies using scanning electron microscopy in order to determine the types of proboscis sensilla in different species and their different feeding habits.

## Materials and methods

### Insects

Voucher specimens representing all sampled species were deposited in the Entomological Museum of Northwest A&F University. Specimen information and location data are presented in Table 1.

**Table 1:** Material localities and collection dates.

Family	Subfamily	Species	Feeding	Localities	Quantity	Voucher
Papilionidae	Papilioninae	*Iphiclides podalirius* (Linnaeus)	nectar	Yili City, Xinjiang Uygur Autonomous Region	10	L0011-L0020
Hesperiidae	Hesperiinae	*Parnara guttata* (Bremer et Grey)	nectar	Yuanyang County, Yunnan Province	10	L4873-L4882
Pieridae	Coliadinae	*Colias fieldii* Ménétriēs	nectar	Xunhua County, Qinghai Province	10	L2353-L2362
Lycaenidae	Polyommatinae	*Celastrina oreas* (Leech)	nectar	Huangling County, Shaanxi Province	10	L3137-L3146
Nymphalidae	Nymphalinae	*Sasakia charonda* (Hewitson)	sap	Taibai Mountain, Shaanxi Province	10	L1177-L1186
	Danainae	*Tirumala limniace* (Cramer)	nectar	Kunming City, Yunnan Province	10	L3921-L3930
	Heliconiinae	*Acraea issoria* (Hübner)	nectar	Wugong County, Shaanxi Province	10	L1589-L1598
	Satyrinae	*Stichophthalma neumogeni* Leech	fruit	Diaoluo Mountain, Hainan Province	10	L2330-L2339
		*Callerebia suroia* Tytler	nectar	Liuba County, Shaanxi Province	10	L2017-L2026
	Libytheinae	*Libythea celtis* (Laicharting)	nectar	Taibai Mountain, Shaanxi Province	10	L4313-L4322

### Scanning electron microscopy

The dried proboscis of each of the ten species was removed at the base from the head of pinned specimens using fine-tip forceps. Each proboscis was washed four times in a 70% ethanol solution for 30 seconds each time in an ultrasonic cleaner (KH-250DB; 15°C, 50 Hz). After dehydration in a graded ethanol series, the samples were freeze-dried in tertiary butanol. They were attached to a holder using electric adhesive tape, sputter coated with gold, and examined and photographed in a Hitachi S-4800 scanning electron microscope (Hitachi, Tokyo, Japan) at 15 kV. Sensillum types were identified according to the description given in Faucheux [20].

### Measurements and statistical analyses

Proboscises and sensilla were measured and counted for ten individuals of each species. The lengths of the proboscises, sensilla chaetica, sensilla basiconica, and sensilla styloconica were measured using a digitizing tablet and the Imaris 7.2.3 software. The mean number and standard deviation were calculated using the Predictive Analytics Software Statistics 20.0 (SPSS Inc., Chicago, IL, USA). Differences in length and diameter of the sensilla were evaluated and compared using an independent sample t-test (P<0.05) in the SPSS 20.0 Statistical Software.

## Results

### General morphology of the proboscis

**Fig 1:**
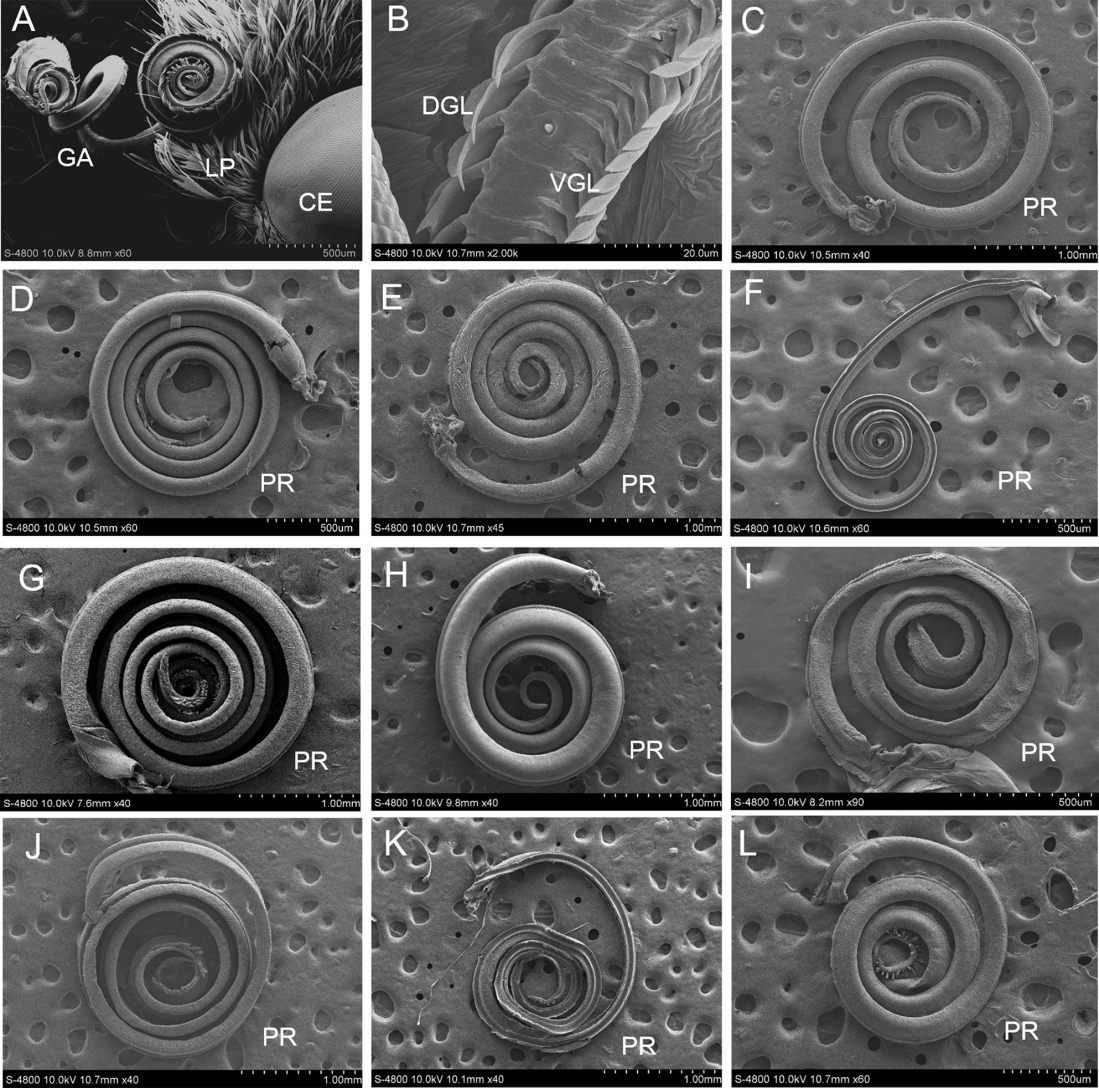
Morphology of proboscis. A: Proboscis (PR) coiled between the labial palpi (LP); CE: compound eye; GA: galea; LP: labial palpus. B: Inner view of the proboscis, showing the dorsal (DGL) and ventral galeal linkage (VGL). C-L: The proboscis. C: *I*. *podalirius*; D: *P*. *guttata*; E: *Co*. *fieldii*; F: *Ce*. *oreas*; G: *Sa*. *charonda*; H: *T*. *limniace*; I: *A*. *issoria*; J: *St*. *neumogeni*; K: *Ca*. *suroia*; L: *L*. *celtis*.

The mouthparts of the ten species of butterfly adults are typically siphonic, with the proboscis coiled tightly between the labial palps (Fig 1). The proboscis is tapered from the base to the tip, and the outer surface is covered with cuticular processes and various types of sensilla. The shape of the cuticular process is hairy or ridged, and four types of sensilla may be observed: sensilla chaetica, sensilla basiconica, sensilla styloconica, and sensilla coeloconica.

### Sensilla chaetica

**Fig 2:**
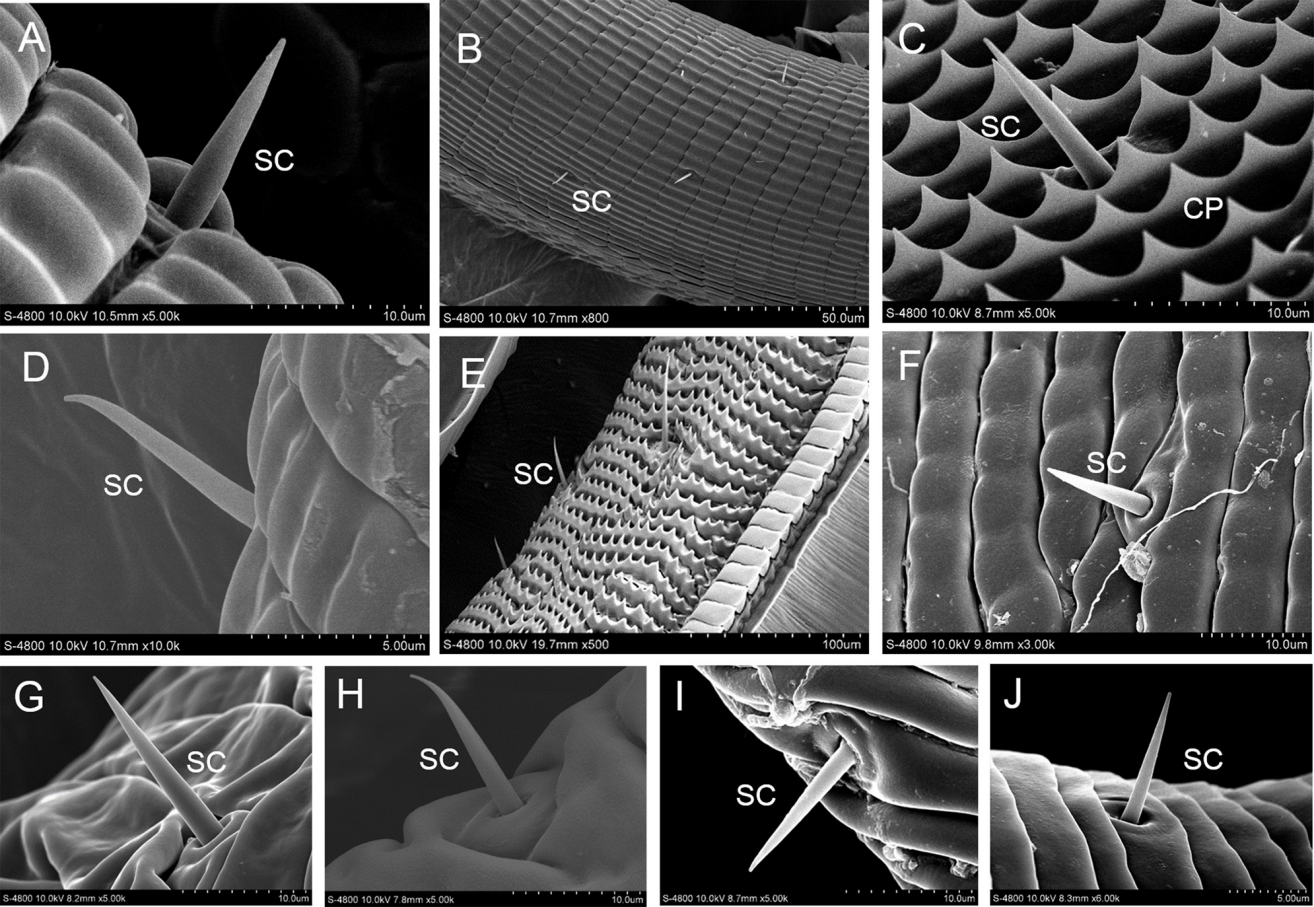
Sensilla chaetica (SC). A: *I*. *podalirius*; B: *P*. *guttata*; C: *Co*. *fieldii*; D: *Ce*. *oreas*; E: *Sa*. *charonda*; F: *T*. *limniace*; G: *A*. *issoria*; H: *St*. *neumogeni*; I: *Ca*. *suroia*; J: *L*. *celtis*.

Sensilla chaetica are distributed on the outer surface of the proboscis and are denser at the base and proximal regions of the proboscis. The bristles of sensilla chaetica are poreless and smooth on the surface of the cuticular wall. Sensilla chaetica are straight or curved at the tapered end and form an angle with the surface of the proboscis (Fig 2).

These sensilla are the most abundant type with about 82–228 per galea in the ten species. The sensilla chaetica gradually become shorter from the proximal part of the proboscis toward the tip and are significantly correlated with the length and diameter (P < 0.01, Table 2).

**Table 2:** Body length, forewing length, proboscis length and length, diameter and number of various sensilla of ten species.

			*I*. *podalirius*	*P*. *guttata*	*Co*. *fieldii*	*Ce*. *oreas*	*Sa*. *charonda*	*T*. *limniace*	*A*. *issoria*	*St*. *neumogeni*	*Ca*. *suroia*	*L*. *celtis*
Body length (mm) n = 10			22.90±0.99	15.20±1.48	20.00±2.21	11.30±0.95	36.30±1.25	30.20±1.62	22.30±1.49	27.70±0.82	19.30±1.34	16.30±1.57
Forewing length (mm) n = 10			38.30±1.57	15.80±0.92	28.10±1.97	16.10±1.29	47.10±1.52	46.60±4.01	35.50±2.95	43.30±1.89	30.90±1.10	22.90±2.69
Proboscis length (mm) n = 10			10.32±0.69	12.17±1.73	10.56±0.92	5.03±0.48	18.63±1.97	9.26±1.15	4.37±0.86	13.96±1.03	8.98±0.55	6.53±0.74
Relative proboscis length			0.45±0.01	0.80±0.05	0.53±0.02	0.44±0.02	0.51±0.04	0.31±0.03	0.19±0.03	0.50±0.02	0.47±0.01	0.40±0.01
Sensilla chaetica	Proximal n = 20	Length (μm)	10.86±1.72	38.18±7.32	7.10±1.34	6.90±1.02	10.35±4.16	9.92±2.70	13.55±5.05	12.28±3.73	10.64±2.67	7.94±1.79
		Diameter (μm)	2.35±0.35	2.49±0.41	1.60±0.25	1.02±0.17	2.49±0.21	1.83±0.55	1.91±0.31	2.08±0.21	1.72±0.26	1.29±0.20
	Mid n = 20	Length (μm)	11.11±2.64	8.34±2.11	7.75±1.54	5.63±0.83	9.12±2.50	6.88±0.84	12.82±3.26	9.85±3.13	11.94±1.79	7.01±1.07
		Diameter (μm)	2.33±0.52	0.99±0.07	1.87±0.30	1.02±0.16	2.30±0.21	1.49±0.27	1.97±0.39	1.85±0.33	1.87±0.32	1.34±0.23
	Tip n = 20	Length (μm)	7.95±1.52	7.90±1.72	8.15±2.11	5.77±0.98	7.73±3.26	6.27±1.06	9.17±3.36	7.21±3.75	11.41±1.96	6.40±1.23
		Diameter (μm)	1.79±0.42	1.03±0.09	1.84±0.34	1.07±0.18	2.33±0.25	1.37±0.17	1.83±0.24	1.85±0.78	1.87±0.21	1.33±0.15
	R n = 60		0.667**	0.913**	0.583**	0.506**	0.470**	0.752**	0.558**	0.592**	0.412**	0.335**
	Number per galea		82	176	109	124	135	221	119	143	199	228
External sensilla basiconica	Proximal n = 20	Length (μm)	4.26±1.97	4.01±0.79	3.34±1.34	2.17±0.66	4.74±1.36	3.07±1.03	5.18±1.78	3.89±2.20	3.78±1.77	3.61±1.70
		Diameter (μm)	2.58±0.33	0.74±0.22	1.70±0.42	1.01±0.12	3.16±0.45	1.85±0.73	2.02±0.48	2.09±0.36	1.55±0.21	1.60±0.46
	Mid n = 20	Length (μm)	6.39±1.34	3.37±1.50	2.86±0.81	2.59±0.99	5.63±1.26	4.28±1.16	4.44±2.45	4.56±1.16	3.54±2.08	2.62±0.87
		Diameter (μm)	2.56±0.32	0.93±0.39	1.83±0.39	1.04±0.22	3.25±0.47	1.28±0.19	1.67±0.36	2.44±0.48	1.61±0.23	1.47±0.27
	Tip n = 20	Length (μm)	7.15±1.27	4.05±1.70	4.16±1.11	2.61±1.12	6.58±1.86	3.64±1.34	5.14±2.78	5.37±2.30	3.99±1.98	2.77±0.99
		Diameter (μm)	2.34±0.40	1.50±0.51	1.94±0.39	1.13±0.22	3.47±0.58	1.83±0.80	2.92±1.91	2.62±0.75	1.84±0.37	1.70±0.35
	R n = 60		0.308*	0.349**	0.472**	0.395**	0.342**	0.343*	0.515**	0.792**	0.316*	0.369**
	Number per galea		38	49	57	85	69	114	37	24	76	95
Internal sensilla basiconica	Length (μm) n = 10		6.82±3.07	5.63±1.51	8.06±1.93	3.66±2.22	18.37±4.83	6.75±0.84	5.10±2.02	7.41±2.81	4.11±1.53	6.73±3.97
	Diameter (μm) n = 10		2.64±0.34	1.79±0.27	2.30±0.32	1.13±0.22	4.57±0.47	2.73±0.57	2.19±0.58	3.14±0.65	2.15±0.45	2.11±0.38
	R n = 10		0.096	0.019	0.105	0.24	0.345	0.245	0.465	0.670*	0.563	0.235
	Number per galea		25	20	23	19	14	35	21	39	34	32
Sensilla styloconica n = 20	Length (μm) n = 20		–	9.97±4.73	10.81±1.36	13.36±2.75	87.62±19.51	–	14.68±4.52	88.52±20.08	24.09±3.37	35.66±8.93
	Diameter (μm) n = 20		–	5.59±0.99	6.90±0.79	6.46±1.26	28.44±5.30	–	9.46±3.46	29.68±5.67	11.41±1.28	10.45±1.48
	R n = 20		–	0.116	0.101	0.139	0.276	–	0.856**	0.426	0.091	0.393
	Number per galea		–	13	24	25	209	–	19	85	25	74
Sensilla coeloconica	Diameter (μm) n = 30		6.03±1.16	–	–	–	–	4.09±0.42	–	–	–	–
	Number per galea		80	–	–	–	–	45	–	–	–	–

Data are presented as Mean ± SE; n: sample size; -: not found; R: pearson correlation coefficient of sensillum length and diameter.

Relative proboscis length is the proboscis length divided by the body length.

Data followed by * and ** are significantly different at P < 0.05 and P < 0.01, respectively.

### Sensilla basiconica

**Fig 3:**
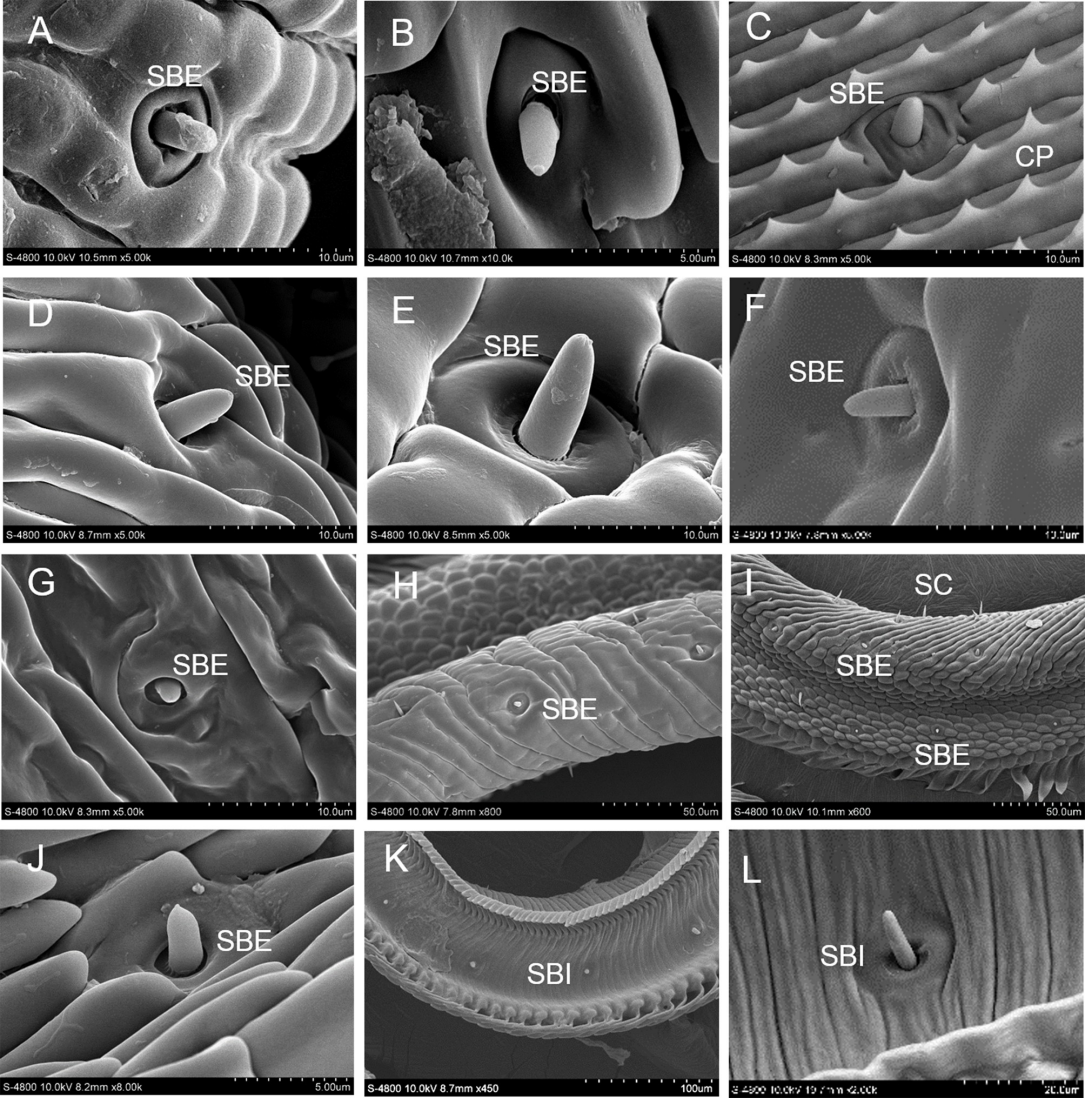
Sensilla basiconica (SB). A-J: An external sensillum basiconicum (SBE). A: *I*. *podalirius*; B: *P*. *guttata*; C: *Co*. *fieldii*; D: *Ce*. *oreas*; E: *Sa*. *charonda*; F: *T*. *limniace*; G: *A*. *issoria*; H: *St*. *neumogeni*; I: *Ca*. *suroia*; J: *L*. *celtis*; K: an internal sensillum basiconicum (SBI) in the food canal; L: magnification of SBI.

Sensilla basiconica are found on the proboscis of ten species, and there are no morphological differences. Each sensilla basiconica is composed of a dome or peg-shaped sensory cone of various lengths with a smooth surface and a single terminal pore, surrounded by a flexible and shallow socket. Sensilla basiconica can be divided into external sensilla basiconica (SBE) existing on the external face of the galeae and internal sensilla basiconica (SBI) existing on the internal face of the food canal. External sensilla basiconica are arranged in irregular rows throughout the dorsal and lateral sides of the proboscis, while internal sensilla basiconica form a single row in the food canal on each galea (Fig 3).

Internal sensilla basiconica (about 14–39 per galea) are longer and less numerous than the external sensilla basiconica (about 24–114 per galea). The lengths of the external sensilla basiconica are positively correlated with their diameters (Pearson correlation coefficient: 0.308–0.792). Except for *St*. *neumogeni*, there are no significant correlations between the lengths and diameters of the internal sensilla basiconica of the other nine species (Table 2).

### Sensilla styloconica

**Fig 4:**
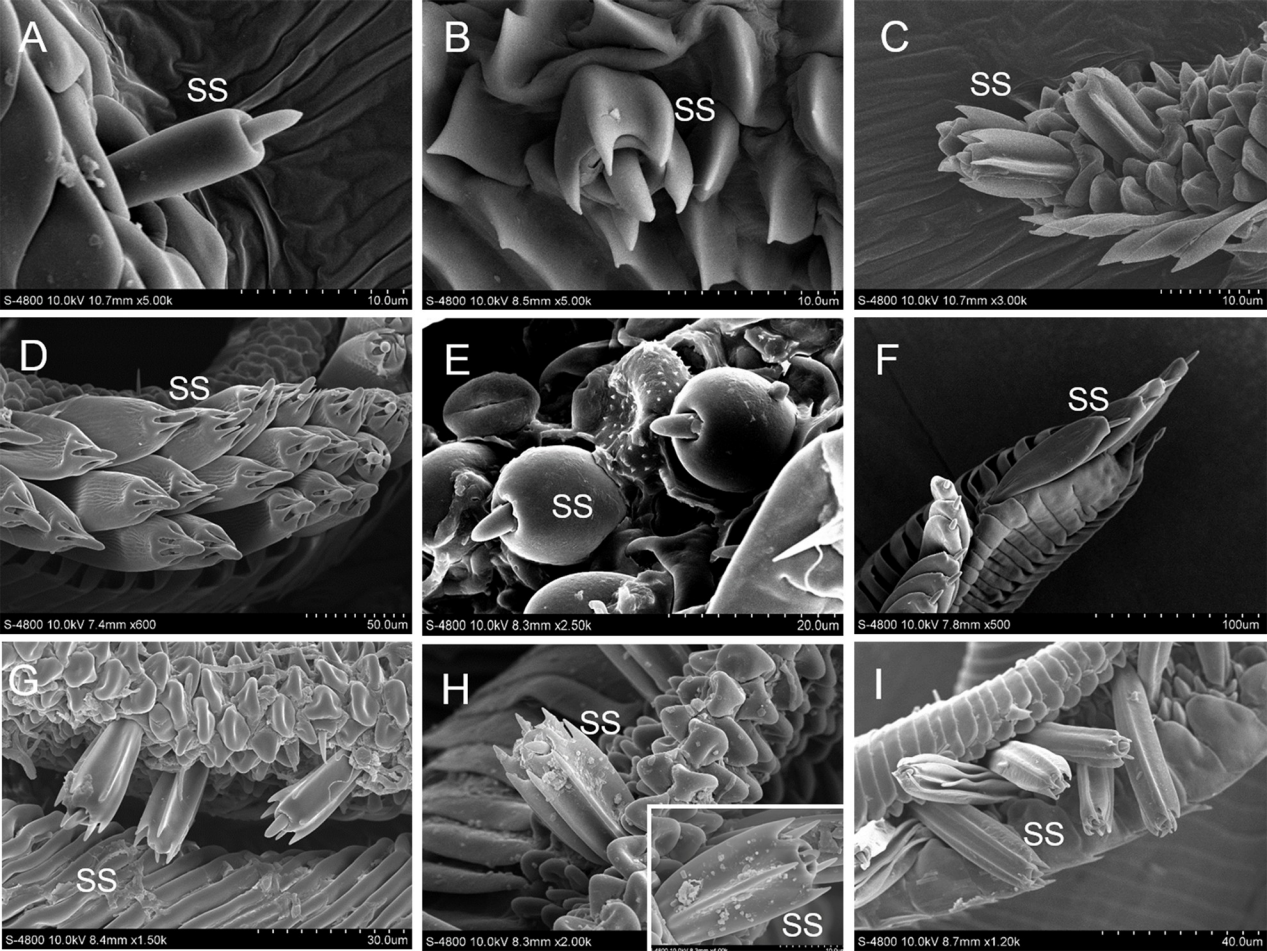
Sensilla styloconica (SS). A: *P*. *guttata*; B: *Co*. *fieldii*; C: *Ce*. *oreas*; D: *Sa*. *charonda*; E: *A*. *issoria*; F: *St*. *neumogeni*; G: SS with six longitudinal ribs of *Ca*. *suroia*; H: SS with five and seven longitudinal ribs of *Ca*. *suroia*; I: *L*. *celtis*.

Sensilla styloconica are found in all but two species, *I*. *podalirius* and *T*. *limniace*. They are confined to the tip region of the proboscis and are regularly arranged in 1 to 3 columns, each consisting of a variously-shaped stylus and a short apical sensory cone. The differences in the sensilla styloconica on the proboscis of the eight species, *P*. *guttata*, *Co*. *fieldii*, *Ce*. *oreas*, *Sa*. *charonda*, *A*. *issoria*, *St*. *neumogeni*, *Ca*. *suroia* and *L*. *celtis*, are mainly reflected in their lengths and shapes. The stylus can be divided into cylindrical, flat, spherical, and the presence or absence of longitudinal ribs (Fig 4).

The number of sensilla is 13–209 per galea. The length of the sensilla styloconica is significantly correlated with their diameter in *A*. *issoria*. Nonetheless, no significant correlation exists in the sensilla styloconica of the other nine species regarding length and diameter (Table 2).

### Sensilla coeloconica

**Fig 5:**
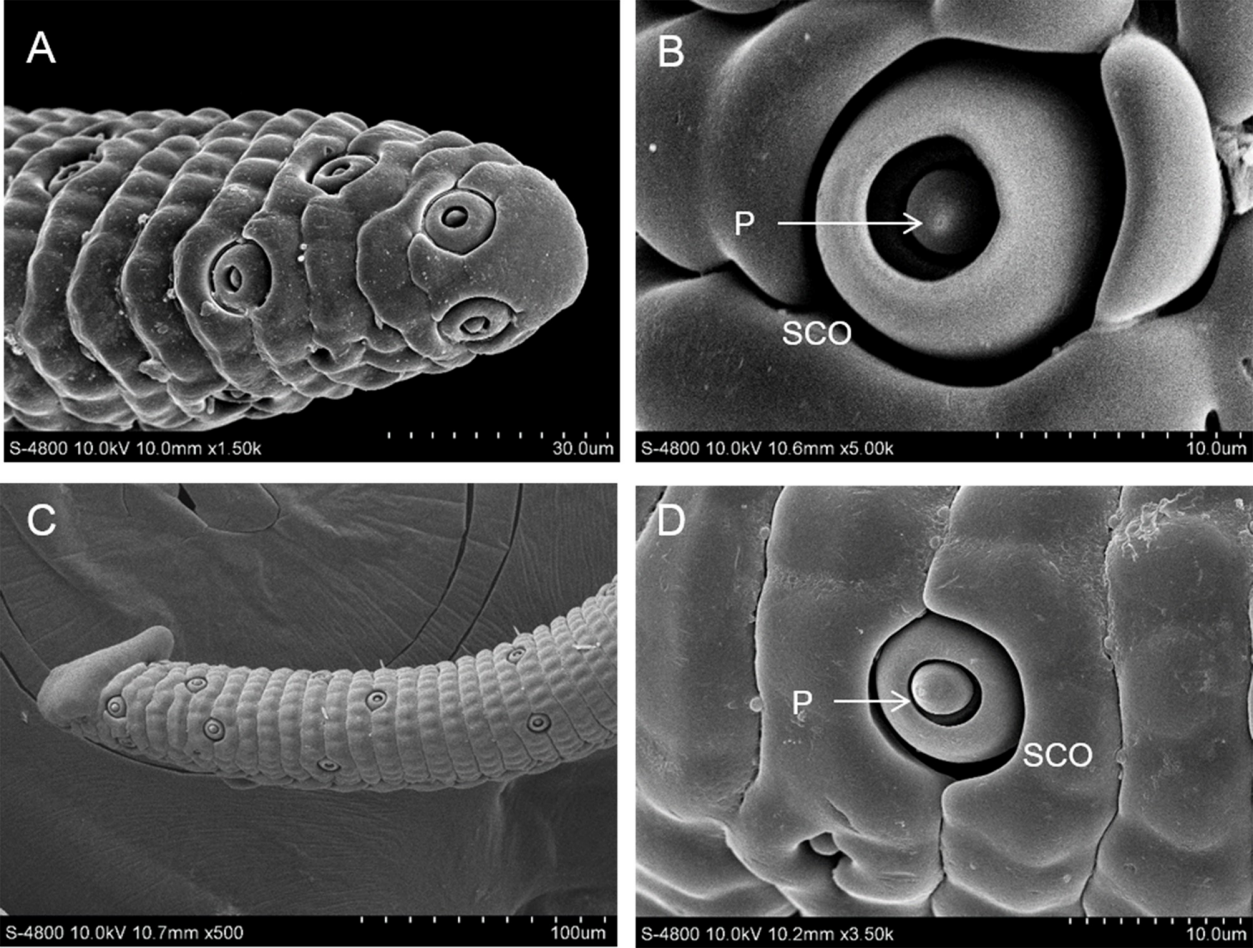
Sensilla coeloconica (SCO). A, B: SCO on the tip region of *I*. *podalirius*; C, D: SCO on the tip region of *T*. *limniace*.

Sensilla coeloconica are observed only in *I*. *podalirius* and *T*. *limniace* and are mainly distributed at the tip of the proboscis. The sensillum coeloconicum has a glabrous and terminal pore sensory cone, located in the annular deep cavity of the galeal cuticle (Fig 5). The external diameters of the cavities of *I*. *podalirius* and *T*. *limniace* are 6.03±1.16 μm (n = 30) and 4.09±0.42 μm (n = 30), respectively. Each galea has approximately 80 and 45 sensilla, respectively, for *I*. *podalirius* and *T*. *limniace*.

## Discussion

For the ten species studied, the shape of the proboscis is consistent with that of other Lepidoptera. The proboscis has a variable length and tapers toward the tip, while the diameter of the food canal remains almost unchanged [20, 32]. Compared with the flower-visiting species, the proboscis of the non-flower-visiting species do not taper as much, and the galeal apex is somewhat rounded. There is a positive correlation between body length and proboscis length both in nectar-feeding and non-nectar feeding butterflies, and nectar-feeders have a tendency to show an allometric relationship [33–36]. The proboscis length of these ten investigated species is between 3.26–22.84 mm, accounting for 16%-90% of body length and 10%-90% of forewing length. The profitability of butterfly foraging depends in part on the corolla depth and clustering of flowers and the tongue length, body mass and wing loading of butterflies [35, 37, 38]. Short-tongued butterflies do not visit flowers with deep corollas, while butterflies with a light wing load generally prefer clustered or nectar-rich flowers [35, 39].

The morphology of sensilla reflects taxonomic relationships and has been used to infer phylogenetic relationships [36, 40, 41]. Proboscis sensilla, morphological differences were found mainly in the length, shape and number of sensilla. According to previous studies, sensilla chaetica, sensilla basiconica, and sensilla styloconica are common types of sensilla in Lepidoptera [1, 20, 21]. In this study, all ten species possess sensilla chaetica and sensilla basiconica on their proboscis, and eight of them also have sensilla styloconica on the proboscis, the exceptions being *I*. *podalirius* and *T*. *limniace*. On the other hand, sensilla coeloconica are only present on the proboscis of *I*. *podalirius* and *T*. *limniace*. Four types of sensilla have been reported in other lepidopterans, such as Nymphalidae [22, 28, 30], Micropterigidae [1], Plusiinae and Noctuinae [14, 16].

Sensilla chaetica differed among the examined species mainly in length and number. It becomes shorter toward the tip of the proboscis, especially in *P*. *guttata*. Previous studies have suggested that this should be an adaptation to feeding, and the width and depth of the tube can be assessed without blocking the entrance for the proboscis during feeding [1, 21]. Compared with flower-visiting species, the bristle-shaped sensilla chaetica are less developed in those species that do not feed on pollen [5]. These sensilla on the flower-visiting species are generally regarded as mechanosensitive and may provide feedback concerning the pollen load, shape and size [15, 21]. However, on the non-flower-visiting species these sensilla are considered to be gustatory organs for host location [42].

The uniporous sensilla basiconica occur both on the external galea and in the food canal, differing only in the length of their sensory cones. Sensilla basiconica with a single terminal pore on the external galea are chemosensilla [13, 21], with those in the food canal functioning as taste receptors [24]. Multiporous sensory cones with olfactory or gustatory functions are found in moths [20, 23, 43–45] but not in the butterflies in this study.

Sensilla styloconica are located at the tip region of the proboscis. Numbers and lengths of sensilla styloconica vary among species. These sensilla can be divided into a uniporous structure as a chemo-mechanoreceptors [21, 27, 46], and a multiporous structure with an olfactory role [47]. In this study, all investigated sensilla styloconica showed only one terminal pore, so the multiporous sensilla styloconica appear to be missing. For the non-flower-visiting species, the number of sensilla styloconica on the proboscis of *Sa*. *charonda* and *St*. *neumogeni* is relatively larger than those of the rest. Kreen et al. [48] studied 64 nymphalid species and found that the investigated non-flower-visiting species also had a greater number of club-shaped sensilla styloconica in the tip region. Alcohol can be detected by these sensilla in a butterfly that feeds on rotting fruit [28]. In this study, we found that these sensilla were absent in *I*. *podalirius* and *T*. *limniace* and are also completely absent in some insects in the genera *Papilio* and *Danaus*, and the families Mnesarchaeidae, Hepialidae, Nepticulidae and Adelidae [5, 20].

Sensilla coeloconica are found only on the proboscis of *I*. *podalirius* and *T*. *limniace*. These sensilla are more commonly found on the proboscis of moths and are associated with the particular food-seizing techniques of certain species [20]. The sensilla coeloconica on the antennae may be humidity sensilla [49, 50] and be involved in the selection of oviposition sites [51].

The extremely elongate mouthparts in insects evolved as adaptations for gaining access to food resources [34, 52–54]. Studies on the relative proboscis length of the family Hesperiidae have inferred that at least three major independent evolutionary events have occurred in this family [33]. In this study, the family Hesperiidae also had the highest relative proboscis length (mean = 0.80), further demonstrating that the proboscis in the family Hesperiidae may have features that independently evolved. In addition, the sensilla are mapped to the cladogram of butterflies [55]. Combined with previous studies, there are sensilla chaetica, sensilla basiconica and sensilla styloconica on the proboscis of six families (except for the family Hedylidae where there is no report about the sensilla on the proboscis) [33, 36, 53]. Therefore, the possible apomorphies of the proboscis in butterflies are these three sensilla. The presence of sensilla coeloconica is inferred as an autapomorphy of *Iphiclides* and *Tirumala*, which remains to be determined.

## Conclusion

We identified four types of sensilla on the proboscis of ten species of butterfly adults. The external morphology and distribution of these sensilla are similar to other reported Lepidoptera. We found differences in the types, numbers, and lengths of sensilla among the species, which may be related to their different hosts and life habits. Our study provides a basis for better understanding of the function of different morphological structures of the proboscis in feeding.
